# Biomass-Derived Porous Carbons Derived from Soybean Residues for High Performance Solid State Supercapacitors

**DOI:** 10.3390/molecules25184050

**Published:** 2020-09-04

**Authors:** Hsiu-Ying Chung, Guan-Ting Pan, Zhong-Yun Hong, Chun-Tsung Hsu, Siewhui Chong, Thomas Chung-Kuang Yang, Chao-Ming Huang

**Affiliations:** 1 Green Energy Technology Research Center and Department of Materials Engineering, Kun Shan University, Tainan 710, Taiwan; hsiuychung@gmail.com (H.-Y.C.); st112926@gmail.com (Z.-Y.H.); 2 Department of Chemical Engineering and Biotechnology, National Taipei University of Technology, Taipei 106, Taiwan; gtpan@ntut.edu.tw; 3 Green Energy & Environment Research Labs, Industrial Technology Research Institute, Tainan 710, Taiwan; ChunTsungHsu@itri.org.tw; 4 Department of Chemical and Environmental Engineering, University of Nottingham Malaysia, Jalan Broga 43500, Malaysia; Faye.Chong@nottingham.edu.my

**Keywords:** biomass-derived porous carbon, hydrothermal carbonization, soybean residues, supercapacitor, solid state electrolyte

## Abstract

A series of heteroatom-containing porous carbons with high surface area and hierarchical porosity were successfully prepared by hydrothermal, chemical activation, and carbonization processes from soybean residues. The initial concentration of soybean residues has a significant impact on the textural and surface functional properties of the obtained biomass-derived porous carbons (BDPCs). SRAC5 sample with a BET surface area of 1945 m^2^ g^−1^ and a wide micro/mesopore size distribution, nitrogen content of 3.8 at %, and oxygen content of 15.8 at % presents the best electrochemical performance, reaching 489 F g^−1^ at 1 A g^−1^ in 6 M LiNO_3_ aqueous solution. A solid-state symmetric supercapacitor (SSC) device delivers a specific capacitance of 123 F g^−1^ at 1 A g^−1^ and a high energy density of 68.2 Wh kg^−1^ at a power density of 1 kW kg^−1^ with a wide voltage window of 2.0 V and maintains good cycling stability of 89.9% capacitance retention at 2A g^−1^ (over 5000 cycles). The outstanding electrochemical performances are ascribed to the synergistic effects of the high specific surface area, appropriate pore distribution, favorable heteroatom functional groups, and suitable electrolyte, which facilitates electrical double-layer and pseudocapacitive mechanisms for power and energy storage, respectively.

## 1. Introduction

Among the devices used as electrical energy storage systems, supercapacitors are the devices between conventional capacitors and rechargeable batteries and have found wide applications in aircrafts (Airbus 380), consumer electronics, emergency backup powers, and hybrid or electric vehicles [1]. Based on the energy storage mechanism, supercapacitors can be classified as EDLCs, pseudocapacitors, and hybrid supercapacitors composed of an EDLC and a pseudocapacitor [1]. Due to the sluggish reaction kinetics of metal oxides, the major electrode materials, pseudocapacitors have poor cycle stability compared to EDLCs. EDLCs exhibit the high power density, rapid charge–discharge cycle, long cycling life, and safety [2]. However, the low energy density of EDLCs has severely limited its practical applications. Thus, it is worthy to improve the energy density of EDLCs without sacrificing its high power density, which will drive the EDLCs into promising power supply devices to replace secondary batteries.

To overcome the low energy density of EDLCs, the developments of electrode materials and electrolytes of EDLCs need to be addressed. Especially, the electrode materials are the key component for determining the performance. Carbon materials such as activated carbon, carbon black, carbon nanotubes, graphene, etc., are widely used as electrode materials in EDLCs [3]. Most of the commercial activated carbons were produced from petroleum coke and charcoals prepared by direct calcination. This results in small specific surface area and poor pore structure, leading to its poor conductivity and high contact resistance between the activated carbons and the electrolyte, which is not beneficial for energy storage applications. It has been reported that porous carbon-based materials with high specific surface area, suitable pore structure, and favorable functional groups have the promising applications as the electrode materials for EDLCs and hybrid supercapacitors [2,4]. Besides, the materials’ cost and environmental impacts during the material preparation are vital to the large-scale commercial popularization of supercapacitors.

The development of high energy density with low cost EDLCs can be approached at three main aspects. From low cost materials perspective, the usage of BDPCs for electrode materials of EDLCs has attracted much interest because of low cost, renewable, huge availability, and environmental friendliness of biomass [5,6]. Several reviews summarize that surface heteroatoms including nitrogen, oxygen, sulfur, boron, and phosphorus can improve the surface wettability and charge transfer kinetics of carbons [7,8,9,10]. Soybean, a renewable plant, is abundant with functional groups such as amino, carboxyl, hydroxyl, and sulfate, which could be considered as both sources for carbon and nitrogen. Thus, soybean residues can be considered as an ideal precursor to prepare nitrogen containing carbon for EDLCs, and it also could be a green and renewable way to convert this vast waste resource into value-added products [11].

For the second perspective, it is highly important to use a cost-effective approach for producing BDPCs from a variety of biomass precursors. Traditionally, the pyrolysis carbonization process, at a temperature between 400 and 850 °C in the absence of oxygen, was applied to prepare activated carbons. Recently, the usage of hydrothermal carbonization for the conversion of a wide variety of biomass into carbon materials has been reported [11,12]. Hydrothermal carbonization method uses the subcritical water to convert the wet/dry biomass to the hydrochar with high oxygenated functional group content, which operated at the temperature range of 150–350 °C within an enclosed vessel. Generally, the hydrochar products of hydrothermal carbonization would be further activation treated to be BDPCs. Relatively low operation temperature and no need of dry biomass mean hydrothermal carbonization of biomass is an environmentally friendly way to prepare low-cost BDPCs.

According to the equation E = 0.5 CV^2^, the energy density of electrochemical cell is proportional to the specific capacitance and the square of the working voltage. However, the enhancement of the specific capacitance cannot be improved significantly due to the double-layer capacitance mechanism of EDLCs [13]. Thus, the last perspective for increasing the energy density is to broaden the working voltage window, which can be achieved by using a suitable electrolyte. From an appropriate electrolyte perspective, the gel electrolyte, between a liquid and a solid, shows promising advantages such as no problem for leakage, high ionic conductivity, and better electrolyte–electrode interaction.

In this work, two approaches were taken to produce low-cost BDPCs as the highly effective electrode materials for high energy EDLCs. The first approach was the usage of soybean residues to make nitrogen-containing hierarchical porous carbons with high specific surfaces. By tuning of soybean residues concentrations (10%, 7%, and 5%) in hydrothermal carbonization and maintaining KOH/hydrochar mass ratio of 1 in chemical activation, the carbonized soybean residue activated carbons (SRACs) exhibited specific surfaces of 1351, 1476, and 1945 m^2^ g^−1^, respectively, for SRAC10, SRAC7, and SRAC5. The optimized SRAC5 coated on Ni foam as the SRAC5/Ni electrode delivered a high specific capacitance of 489 F g^−1^ at 1 A g^−1^ in 6 M LiNO_3_ aqueous electrolyte. The other approach was the application of a solid-state carboxymethyl cellulose–lithium nitrate (CMC–LiNO_3_) electrolyte. The symmetric SRAC5/Ni//SRAC5/Ni supercapacitor demonstrated a remarkable high specific energy of 67.3 Wh kg^−1^ at a power density of 2.0 kW kg^−1^ at 2 A g^−1^ in a wide voltage window of 0–2.0 V and an excellent 89.9% capacitance retention after 5000 cycles. These facts demonstrate that the use of hydrothermal carbonization/chemical activation process accomplished with a suitable electrolyte can be the green and facile strategies for fabrication large-scale commercial supercapacitors.

## 2. Results

### 2.1. Texture and Morphology Analysis

**Figure 1:**
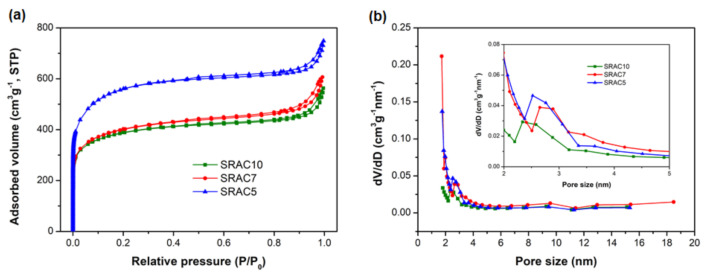
(**a**) Nitrogen adsorption–desorption isotherm curves; and (**b**) pore size distribution of SRACs.

Based on the mechanism of energy store of EDLCs, the supercapacitive performance relies on the interface between the carbon electrode and electrolyte solution. To increase the efficiency of the interface, the textural properties and the wettability of the carbon electrode materials play important roles in determining the supercapacitive performance of EDLCs. Generally, the pore size within carbon electrode materials should be larger than those of the ion size of the electrolyte and the pore size larger than 0.5 nm could be beneficial for access of the ions of aqueous electrolytes [14]. Therefore, the textural analysis was performed to investigate the parameters of specific surface area, pore size and pore volume of as-prepared SRACs. Compared with the Type I isotherm of conventional activated carbons, the N_2_ adsorption–desorption isotherm of the SRACs presented in Figure 1a showed a typical hybrid of type I and type II with H2 hysteresis loop, indicating the coexistence of micro-, meso-, and macropores [15]. A steep uptake of N_2_ at the low relative pressure (*P*/*P*_0_ ≤ 0.01) was observed for all samples, indicating the existence of abundant micropores. A small hysteresis loop (H2) associated with a capillary condensation step at medium and high relative pressure regions (*P*/*P*_0_ = 0.45–0.89) manifests the presence of mesopores. The steep increase at a high relative pressure (*P*/*P*_0_ > 0.9) without an adsorption plateau was observed, suggesting the existence of macropores. Figure 1b shows the corresponding pore size distributions of as-obtained SRACs. It can be seen that all samples are highly porous with a typical hierarchical pore texture. The SRAC10 sample has a narrow pore size distribution centered at about 2.3 nm with a small portion extending to the mesopore scale. When the biomass concentration (dried soybean residue) during thermal carbonization was reduced, the pore size distributions of SRAC7 and SRAC5 showed multimodal structures. From the inset of Figure 1b, there were more mesopores distributing at the range of 2–5 nm of SRAC7 and SRAC5, compared to SRAC10. In particular, the pore size distribution widened and shifted towards larger pores as the biomass concentration decreased. There have recently been a number of reports suggesting that the high surface area along with a suitable mesopore range of 2–8 nm offers the high accessible surface area and channels for ion transportation, resulting in enhancing energy and power densities of EDLCs [14,16,17,18,19].

The effect of biomass concentration on the textural characteristics of SRACs are given in Table 1. The BET specific surface areas of SRAC10, SRAC7, and SRAC5 were 1351, 1476, and 1945 m^2^ g^−1^, respectively. The microporous specific surface area and total pore volume of the SRACs gradually increase from 817 to 1034 m^2^ g^−1^ and from 0.86 to 1.13 cm^3^ g^−1^ with the decrease of biomass concentration, respectively. Obviously, the textural characteristics the SRACs are significantly influenced by the biomass concentration. As seen, decreasing the biomass concentration brought about an increase in the BET surface area, microporous specific surface area, and total pore volume.

**Figure 2:**
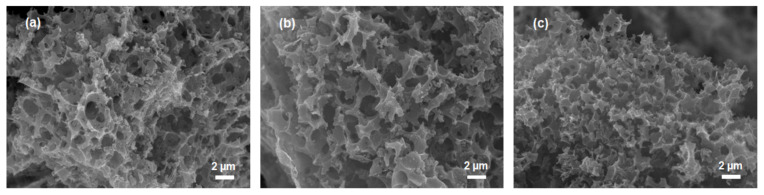
SEM images of: (**a**) SRAC10 powder; (**b**) SRAC7 powder; and (**c**) SRAC5 powder.

Figure 2 illustrates the SEM images of SRACs and rich porous network structure can be clearly observed. SEM images of SRAC10 and SRAC7 showed micrometer-sized corner lines of the hole openings. As shown in Figure 2c, a three-dimensional connected porous structure with sub-micrometer size was obtained for SRAC5. The quantity of sub-micrometer pore increased with decreasing biomass concentration, resulting in a more homogeneous pore structure. Based on the texture and morphology results, it can be concluded that the preparation of high-surface-area activated carbon together with hierarchical porous structure has been successfully developed by tuning biomass concentration in the hydrothermal carbonization/chemical activation process. Hierarchical activated carbons with high surface area can be the promising electrode materials for EDLCs, where macropores promote facile accessibility for the electrolyte and micro-/mesopores provide large accessible spaces for ions.

### 2.2. Structure Analysis

**Figure 3:**
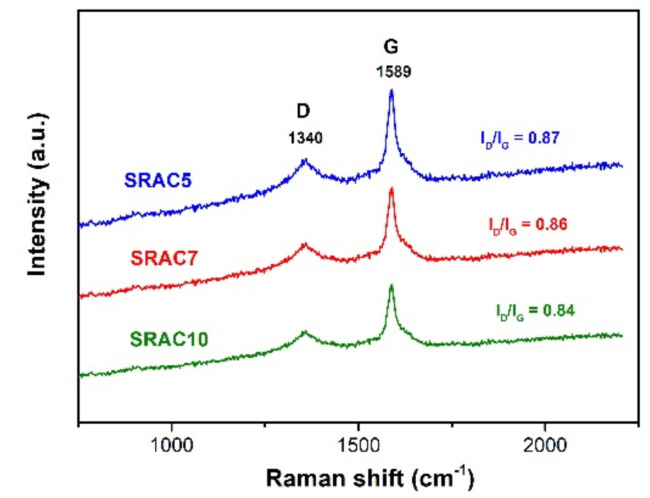
Raman spectra of the as-prepared SRACs.

Raman spectroscopy was employed to analyze the microstructure of the prepared SRACs. Two characteristic Raman peaks are observed distinctly in Figure 3 at about D (1340 cm^−1^) and G (1589 cm^−1^). The D band is commonly assigned to the structure defects and disordered state of activated carbon materials while the G band is associated with the ordered graphitic structure with the stretching vibration of sp^2^-bonded carbon atoms [20,21]. The D to G band integrated intensity ratio (I_D_/I_G_) reflects the degree of the structural ordering with respect to graphitization of carbon materials and a lower value indicates a higher graphitization degree [22]. Here, the I_D_/I_G_ values of the SRACs were in the following order: SRAC5 (0.87) > SRAC7 (0.86) > SRAC10 (0.84). This indicated that low biomass concentration brings about the decrease of the graphitization degree, which may be attributed to higher surface area and larger pore volume, as listed in Table 1.

### 2.3. Composition Analysis

**Figure 4:**
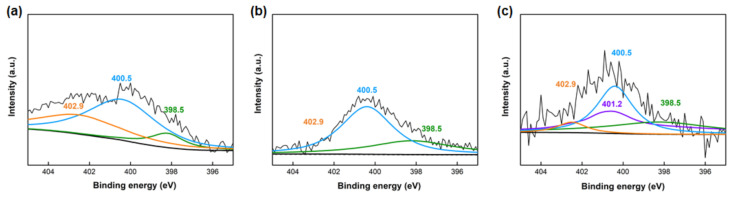
The high-resolution XPS N1s spectra of: (**a**) SRAC10; (**b**) SRAC7; and (**c**) SRAC5.

Surface carbon, oxygen, and nitrogen groups of the as-obtained carbons were investigated by XPS. As listed in Table 1, the SRAC5 has more oxygen and nitrogen species than those of SRAC10 and SRAC7. Owing to the presence of amino groups in soybean, the atomic ratio of nitrogen for the SRACs was about 1–4% and the oxygen content of all samples was high (more than 10 at %) due to the low biomass concentration in hydrothermal activation. Figure 4 shows the high-resolution N1s spectra of samples and the presence of four or three peaks corresponding to pyridinic-N (398.5 eV), pyrrolic-N (400.5 eV), quaternary-N (401.2 eV), and pyridine-N-oxide (402.9 eV) was observed [23,24,25]. Based on the peaks shown in Figure 4, the peak assignments for N1s spectra are summarized in Table 2. As can be seen, reduction of biomass concentration leads to an obvious change in the kind and amount of nitrogen species in the samples. Pyridinic-N and pyrrolic-N are the dominant nitrogen surface functional groups in SRAC7 and SRAC5, with the contents in the range of 34.1–35.3% and 63.9–44.3%, respectively.

### 2.4. Electrochemical Performance of the Electrode

**Figure 5:**
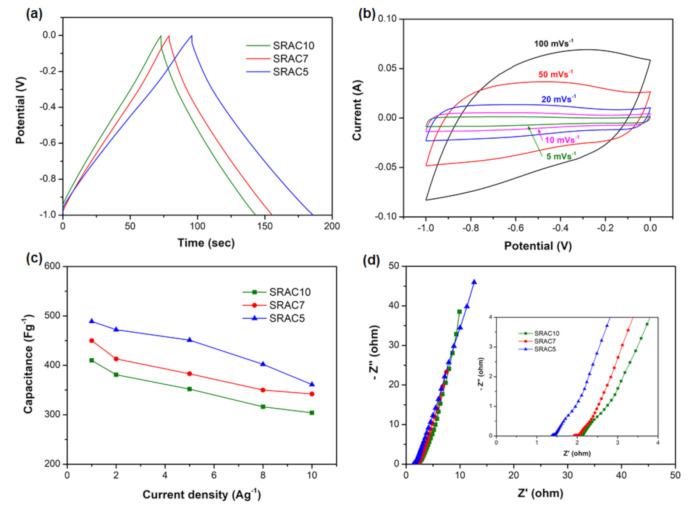
Electrochemical characteristics of SRAC/Ni electrodes in 6 M LiNO_3_ aqueous electrolyte in a three-electrode system: (**a**) GCD curves at 5 A g^−1^ for SRAC/Ni electrodes; (**b**) CV curves of SRAC5/Ni electrode at various scan rates; (**c**) specific capacitances of SRAC/Ni electrodes at various current densities; and (**d**) Nyquist plots of SRAC/Ni electrodes.

To understand the electrochemical performance of SRAC/Ni composites as the active supercapacitor electrodes, GCD, CV, and EIS were carried out in a three-electrode system using a 6 M LiNO_3_ aqueous electrolyte. At the current density of 5 A g^−1^, the specific capacitance values of 352, 383, and 451 F g^−1^ were obtained for SRAC10/Ni, SRAC7/Ni, and SRAC5/Ni, respectively (Figure 5a). The GCD curves showed almost isosceles triangular shapes and slightly deviated from a linear shape, suggesting a certain pseudocapacitive contribution linked to the surface functional groups present in SRACs [26,27]. Figure 5b shows the CV curves of SRAC5/Ni at scan rates between 5 and 100 mV s^−1^, and a pronounced increase of current densities in the potential range of 0 to −1 V was observed, further confirming the existence of substantial faradaic capacitance, originating from the nitrogen and oxygen groups [28]. As shown in Figure 5c, very high values of specific capacitance are obtained at current densities in the range of 1–10 A g^−1^ for the as-prepared samples. The specific capacitance of SRAC5/Ni is the highest among all samples. Its specific capacitance drops from 489 F g^−1^ at 1 A g^−1^ and 451 F g^−1^ at 5 A g^−1^ to 361 F g^−1^ at 10 A g^−1^, revealing the superior electrochemical performance, much higher than most previously reported nitrogen or oxygen-containing carbons [29,30,31]. Figure 5d presents the electrochemical impedance spectra. In the high-frequency region, the intercept with the *x-*axis is the ohmic resistance derived from the internal resistance of electrolyte, the contact resistance between the electrolyte and the electrode, and the intrinsic resistance of electrode (*Rs*). The estimated *Rs* values were 2.2, 1.8, and 1.3 Ω for SRAC10/Ni, SRAC7/Ni, and SRAC5/Ni electrodes, respectively. In medium-high-frequency region, the diameter of the semicircle stands for the charge-transfer resistance (*R_ct_*) at the interface between the electrolyte and electrode. A small semicircle or negligible semicircle was observed, indicating the low faradic charge-transfer resistance. In the low-frequency region, the straight line is the Warburg resistance (*Ws*), responding to the ion diffusion/transport resistance from electrolyte to the surface of electrode. The high slope (about 45°) of the Warburg straight lines reflects a low diffusion resistance of electrolyte ions in SRACs/Ni electrodes.

As described above, the optimal biomass concentration is 5% as the SRAC5/Ni electrode, which has the highest specific capacitance and the lowest ohmic resistance among the samples. The excellent electrochemical performance of SRAC5/Ni might be due to the synergistic effects of hierarchical porosity and the functional groups with heteroatom species (e.g., N and O). Based on the LiNO_3_ aqueous electrolyte, the electrolyte ions could penetrate into the micropores larger than 0.40 nm, since the hydrated diameters are 0.38 and 0.34 nm for Li^+^ and NO_3_^−^, respectively [32]. SRAC5 has the highest BET surface area and high pore density in the mesopore range of 2–4 nm, which provides a high effective surface area and fast ion diffusion, and thus enhances the capacitance. In addition, the high contents of pyridinic-N and pyrrolic-N of SRAC5 could add an extra pseudocapacitance, resulting in a significant increase in the capacitance. Taking into consideration the diffusion kinetics of ions in aqueous electrolyte, the better wettability between the electrolyte and surface pores, the smaller the contact resistance. According to XPS results listed in Table 1, the presence of large amount hydrophilic oxygen and nitrogen species on the SRAC5 surface could increase the hydrophilicity and polarity of an electrode, leading to enhanced wettability and facilitating rapid electrolyte ion transport and reducing the ohmic resistance. Therefore, a rational designed naturally nitrogen-rich BDPCs can not only possess an improved capacitance but also transport the ions more efficiently via the hierarchical porous structure, which fully utilizes electrical double-layer and pseudocapacitive mechanisms for charge storage, respectively.

The yield was defined as the weight ratio of the final AC to initial dried biomass. The yields for SRAC10, SRAC7, and SRAC5 were 20 ± 0.5, 16 ± 0.7, 8 ± 0.6% and respectively. The percent yield of SRACs decreased significantly with an increase of the water amount used in the hydrothermal carbonization process (HTC). The decrease in percent yield can be attributed to the increased aqueous phase. In HTC, biomass will be broken down into small molecules in sub-/super-critical water as aqueous phase and most of the molecules would re-polymerize to form hydrochar. The more water used, the more aqueous phase produced, resulting in the lower percent yield of final AC. SRAC5 presents the best electrochemical performance among the samples; however, the percent yield of SRAC5 is around 8% and too low productivity harms large-scale application. Therefore, the further amount of water used beyond the SRAC5 was not investigated.

### 2.5. Electrochemical Performance of the Symmetric Supercapacitor

**Figure 6:**
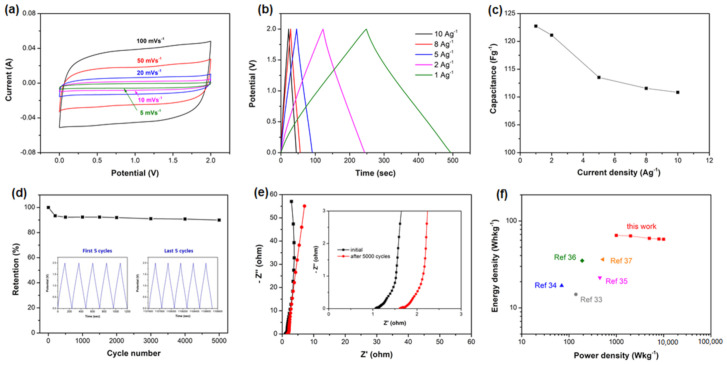
Electrochemical characteristics of the solid-state SRAC5/Ni//SRAC5/Ni supercapacitor: (**a**) CV curves at various scan rates within a voltage window of 2 V; (**b**) GCD curves at various current densities; (**c**) the gravimetric capacitance as a function of current density; (**d**) cycling performance at a current density of 2 A g^−1^ (inset: the charge–discharge curves of the first and last 5 cycles); (**e**) Nyquist plots of the initial and after 5000 cycles; and (**f**) Ragone plots comparison of the SRAC5/Ni//SRAC5/Ni SSC versus previously reported soybean-based SSC devices.

Based on the findings of a single electrode study, a SSC device (SRAC5/Ni//SRAC5/Ni) was assembled using two identical SRAC5/Ni electrodes in solid-state CMC–LiNO_3_ gel electrolyte. Figure 6a displays the CV curves of the SRAC5/Ni//SRAC5/Ni SSC at scan rates ranging from 5 to 100 mV s^−1^ over a voltage window of 2 V. They all exhibit a unique quasi-rectangular shape without obvious distortions, indicating an ideal EDLC nature during the charge–discharge process. The rectangular shape is well retained even at the high scan rate of 100 mV s^−1^, suggesting fast charge–discharge rates and low equivalent series resistance. The GCD measurement was conducted at various current density from 1 to 10 A g^−1^ between 0 and 2.0 V, as shown in Figure 6b. The charge curves are nearly symmetric with the corresponding discharge counterparts, further confirming the dominated double layer charge storage effects. Figure 6c showed the mass specific capacitances of the SSC at 1, 2, 5, 8, and 10 A g^−1^ were 123, 121, 114, 112, and 111 F g^−1^, respectively, where the used mass loading (about 3.6 mg cm^−2^) is based on the total mass of the active materials in both electrodes. The cycling stability of the SSC was evaluated at a current density of 2 A g^−1^ between 0 and 2.0 V, as displayed in Figure 6d. It can be found that the assembled SSC device has excellent cycling durability with 89.9% retention after 5000 successive GCD cycles and there is no obvious difference observed for the first and the last five charge–discharge cycles. Moreover, the EIS test was employed to analyze the resistance change after the cycling test. As presented in Figure 6e, the *Rs* value of the device just increased a little from 1.1 to 1.6 Ω after 5000 cycles, and the steep Warburg line was retained after long-term cycling, exhibiting remarkable stability. The energy density (*E*) and power density (*P*) of the prepared SSC device were calculated based on the GCD curves using Equations (1) and (2) and the Ragone plots of the energy density and the power density of SRAC5/Ni//SRAC5/Ni SSC is shown in Figure 6f. It is notable that SRAC5/Ni//SRAC5/Ni SSC delivered a maximum energy density of 68.2 Wh kg^−1^ at the power density of 1 kW kg^−1^ with a voltage window of 2 V. More impressively, the energy density still can be kept at 61.6 Wh kg^−1^ even at a power density as high as 10 kW kg^−1^. For the sake of comparison, the preparation process, electrolyte, and electrochemical performance of recently developed soybean-based SSC devices are listed in Table 3 and the energy density and the power density of the mentioned literature are also shown in Figure 6f. Obviously, *E* and P in the present study are superior to those of most previously reported soybean-derived SSC devices [33,34,35,36,37]. All these impressive electrochemical performances of the fabricated SRAC5/Ni//SRAC5/Ni SSC may be ascribed to the synergistic effects of high surface area, proper pore distribution, favorable heteroatom functional groups, and the type of electrolyte, which offer sufficient sites for charge storage, channels for fast ion transport, and the increase of working voltage window, leading to the superior electrochemical performance.

## 3. Materials and Methods

### 3.1. Chemicals and Materials

Soybean residues were obtained from local soymilk drink factory. Ni foam was supplied by Green Energy and Environment Research Laboratories of Industrial Technology Research Institute. All reagents (analytical grade) were purchased from Ming Yuh Sci. Instru. Ltd. (Tainan, Taiwan) and used without purification.

### 3.2. Fabrication of SRAC

Soybean residues were chosen as the raw materials for the preparation of BDPCs because not only they are low cost and easily obtained but also they are naturally nitrogen-rich. Prior to use, the soybean residues were washed with water, dried in an oven at 110 °C for 8 h, and then grounded into powder by a grinder. To investigate the effect of the biomass concentration, 6 g of dried soybean powder and a certain volume of water (54, 80, and 114 mL) were mixed together. The as-prepared mixture was poured into a 250 mL Teflon-lined stainless-steel autoclave followed by hydrothermal carbonization treatment at 180 °C for 12 h. Subsequently, the autoclave was cooled to room temperature and the obtained hydrochars were dried at 90 °C overnight. Afterward, the KOH activation was performed as follows: the dried hydrochars were blending with KOH at a weight ratio of 1:1 and dispersed in 40 mL DI water, then under stirring at 90 °C for 2 h and evaporated at 80 °C. Next, the carbonization of hydrochars/KOH mixture was performed in a tubular furnace and then heated to 800 °C for 4 h under N_2_ flow with a flow rate of 80 mL min^−1^. Thereafter, the carbonized carbon was washed with diluted HCl and deionized water several times until the solution pH was neutral. Finally, the resulting activated carbons were vacuum-dried at 70 °C overnight. The as-obtained products were denoted as SRAC10, SRAC7, and SRAC5 based on the initial biomass concentration, respectively.

### 3.3. Materials Characterization

The morphology of samples was characterized using field emission scanning electron microscopy (JEOL, JSM-6700F, Tokyo Japan). The surface elemental composition of as-prepared carbons was characterized by X-ray photoelectron spectroscopy (JEOL, JPS-9030, Tokyo Japan) and the XPS spectra were calibrated by the C 1 s at 284.6 eV. The Raman spectrum was recorded by a micro Raman spectrometer (UniNanoTech, ACRON, Suwon City, Korea) with 532 nm laser excitation. Textural properties of the obtained activated carbons were analyzed using N_2_ adsorption–desorption analyzer (Micromeritics, ASAP 2020A, Norcross, GA, USA) The surface area was calculated by the Brunauer–Emmett–Teller (BET) model based on the adsorption branch in the relative pressure range *P*/*P*_0_ < 0.3. The total pore volume, V_t_, was estimated from the amount adsorbed at *P*/*P*_0_ = 0.995 while the micropore volume, V_micro_, was determined using the t-plot method. The pore size distributions were determined by applying nonlocal density functional theory to the adsorption data for slit pores.

### 3.4. Preparation of Electrodes and Assembly of Symmetric Supercapacitor

The working electrodes, SRAC/Ni, were prepared by a slurry coating process and the slurry was composed of 75 wt% SRAC, 5 wt% KS-6, 15 wt% Super P, and 5 wt% polyvinylidene fluoride binder dissolved in N-methyl-2-pyrrolidinone solvent [38]. The resultant slurry was uniformly pasted on the pre-cleaned Ni foam (3.2 cm^2^), and then dried at 80 °C for 8 h in a vacuum oven. The mass loading of the active materials in each working electrode was about 1.8 mg cm^−2^. The symmetric supercapacitors were fabricated by utilizing two identical working electrodes with approximately the same mass, a CMC–LiNO_3_ gel electrolyte, a separator (polypropylene sheet), and a package material (antistatic aluminum foil). CMC–LiNO_3_ gel electrolyte was prepared as follows: 1 g of CMC was added in a definite quantity of DI water under vigorous stirring until it became a clear solution. After complete dissolution, 2 g of LiNO_3_ were added in the CMC solution and continuously stirred at 45 °C until it got transparent and viscous appearance [39]. Then, these two working electrodes were coated with CMC–LiNO_3_ gel electrolyte, separated by a polypropylene separator, and packaged in antistatic aluminum foil.

### 3.5. Electrochemical Measurements

The electrochemical measurements were carried out in both three-electrode configuration for electrodes and two-electrode configuration for symmetric supercapacitors. In the three-electrode setup, the SRAC/Ni was as the working electrode, a Pt foil as the counter electrode, a saturated calomel electrode (SCE) as the reference electrode, and 6 M LiNO_3_ aqueous solution as the electrolyte [40]. The cyclic voltammetry (CV), electrochemical impedance spectroscopy (EIS), and galvanostatic charge–discharge (GCD) measurements were performed with an electrochemical workstation (6273E, CH Instruments, USA). The EIS measurements were recorded in the frequency range of 0.01 Hz to 100 kHz. The voltage window is from −1.0 to 0 V vs. SCE for the SRAC/Ni electrodes and thus 0–2.0 V for the symmetric SRAC/Ni//SRAC/Ni supercapacitors. From the GCD curves, the specific capacitance (*C_m_*) of electrode was calculated based on the equation *C_m_* = [(*I* × Δ*t*)/ (*m* × *V*)], where *I*, Δ*t*, *m*, and *V* are the discharging current, the discharging time, the mass of the electroactive materials, and the voltage change, respectively. For symmetric supercapacitors, the specific capacitance was calculated based on the equation *C_SC_* = [(*I* × Δ*t*)/ (*M* × *V*)], where *M* is the total mass of electroactive materials based on two electrodes. The cyclic stability of the symmetric supercapacitor was evaluated by battery testing station (580 Battery Test System, Scribner Associates Inc., Southern Pines, NC, USA) in the voltage window of 0–2.0 V at 2 A·g^−1^.

The specific energy density (E) and specific power density (P) of the symmetric supercapacitor were calculated using Equations (1) and (2):

E = 0.5 CV^2^ (Wh·kg^−1^) (1)

P = E/t (W·kg^−1^) (2)

where C (F g^−1^) is the gravimetric specific capacitance of the symmetric supercapacitor, V (V) is the operating voltage window, and t (s) is the discharge time.

## 4. Conclusions

BDPCs with high specific surface area, hierarchical porosity (micro-, meso-, and macropores), and containing heteroatoms (N and O), were successfully prepared by hydrothermal treatment of soybean residues followed by KOH activation and carbonization. The textural and surface functional properties were influenced by the initial soybean residues concentration while maintaining a constant KOH:hydrochar ratio. The fabricated SRAC5/Ni electrode can achieve a high specific capacitance of 489 F g^−1^ at 1 A g^−1^, much higher than most previously reported BDPCs. It might be due to the synergistic effects of hierarchical porosity and the functional groups with heteroatom species (e.g., N and O). The high surface area along with hierarchical pore structure provides efficient electrolyte accessibility and rapid ion diffusion/transport, resulting in enhancing energy and power densities. The self-containing heteroatom species are beneficial to improve the surface wettability and electrical conductivity, introducing pseudocapacitive behavior and reducing the contact resistance. More impressively, a solid-state symmetric supercapacitor device based on the obtained SRAC5/Ni electrode using CMC–LiNO_3_ gel electrolyte can deliver an excellent energy density of 68.2 Wh kg^−1^ at the power density of 1 kW kg^−1^ at a high operating voltage window of 2.0 V. Furthermore, it also exhibits good cycling stability of 89.9% retention over 5000 cycles. These fantastic performances certify that the green synthesis strategy developed can convert a waste intrinsic nitrogen-containing biomass into BDPCs toward realistic applications in low-cost and high-performance supercapacitors. Considering the diversity of biomass resources, the proposed method in this study is considered as an effective way to control the beneficial physical/chemical characteristics such as large specific surface area, rich pore structure, and adjustable pore size of porous activated carbons for EDLCs.

## Figures and Tables

**Table 1:** Textural properties and element components of SRACs.

Sample	Textural Properties					XPS (atom %)		
	S_BET_ (m^2^ g^−1^)	S_mic_ (m^2^ g^−1^)	V_t_ (cm^3^ g^−1^)	V_mic_ (cm^3^ g^−1^)	D_ave_ (nm)	C	N	O
SRAC10	1351	817	0.86	0.37	2.55	85.0	1.3	13.7
SRAC7	1476	809	0.94	0.36	2.35	85.3	1.6	13.1
SRAC5	1945	1034	1.13	0.47	2.34	80.4	3.8	15.8

S_BET_, total BET specific surface area; S_mic_, microporous specific surface area; V_t_, total pore volume; V_mic_, microporous volume; D_ave_, average pore size.

**Table 2:** Peak assignment of N 1*s* of SRACs.

Sample	Nitrogen Functional Groups (%)			
	Pyridinic-N (398.5 ± 0.2 eV)	Pyrrolic-/Pyridonic-N (400.5 ± 0.3 eV)	Quaternary-N (401.2 ± 0.2 eV)	Pyridine-N-Oxide (402.9 ± 0.4 eV)
SRAC10	10.3	51.7	3.5	34.5
SRAC7	34.1	63.9	-	2.00
SRAC5	35.3	44.3	12.7	7.7

**Table 3:** Comparison of energy storage performance of soybean-based activated carbons.

Preparation Process	Electrolyte	Potential Window	Energy Density	Cycling Stability	Reference
Thermal-treatment of KOH-soaked soybean	1 M Na_2_SO_4_ aqueous solution	1.8 V	14.3 Wh kg^−1^	-	[33]
Hydrothermal treatment, KOH activation and then carbonization	1 M Li_2_SO_4_ aqueous solution	1.7 V	18 Wh kg^−1^ (at 0.2 Ag^−1^)	10,000 cycles: 90–95% retention	[34]
Hydrothermal treatment, KOH activation and then carbonization	1 M Na_2_SO_4_ aqueous solution	2.0 V	22.28 Wh kg^−1^ (at 0.5 Ag^−1^)	10,000 cycles: 91.1% retention at 5 Ag^−1^	[35]
Hydrothermal and then carbonization	1 M Na_2_SO_4_ aqueous solution	1.9 V	35 Wh kg^−1^ (at 0.1 Ag^−1^)	2000 cycles: 81.4% retention at 0.5 Ag^−1^	[36]
Hydrothermal treatment, carbonization and then KOH activation	6 M KOH aqueous solution	1.0 V	36.11 Wh kg^−1^ (at 0.5 Ag^−1^)	10,000 cycles: 87.5% retention at 10 Ag^−1^	[37]
Hydrothermal treatment, KOH activation and then carbonization	CMC–LiNO_3_ gel electrolyte	2.0 V	68.19 Wh kg^−1^ (at 1.0 Ag^−1^)	5000 cycles: 89.9% retention at 2 Ag^−1^	This work
